# Less endocrine therapy in HR+/HER2- breast cancer: a nationwide trend despite unchanged guidelines

**DOI:** 10.1016/j.breast.2025.104664

**Published:** 2025-11-26

**Authors:** Eline E.F. Verreck, Emily L. Postma, Tanja Oostergo, Joyce Meijer, Anouk Eijkelboom, Sabine Siesling, Dimitris Rizopoulos, Thijs van Dalen, José H. Volders

**Affiliations:** a Department of Surgery, Diakonessenhuis, Utrecht, the Netherlands; b Department of Surgery, Erasmus Medical Centre, Rotterdam, the Netherlands; c Department of Surgery, St. Antonius hospital, Nieuwegein, the Netherlands; d Department Internal Medicine, Diakonessenhuis, Utrecht, the Netherlands; e Department of Health Technology and Services Research, Technical Medical Centre, University of Twente, Enschede, the Netherlands; f Department of Research and Development, Netherlands Comprehensive Cancer Organisation (IKNL), Utrecht, the Netherlands; g Department of Biostatistics, Erasmus Medical Centre, Rotterdam, the Netherlands; h Department of Surgery, University Medical Centre Utrecht, Utrecht, the Netherlands

**Keywords:** Breast cancer, Hormone receptor-positive, Endocrine therapy, Regional variation

## Abstract

**Background:**

Adjuvant systemic therapy has improved breast cancer outcomes over the past decades. Following the distinction of molecular subtypes and the introduction of gene expression profiling and prognostic tools, chemotherapy is less frequently recommended for hormone receptor-positive, HER2-negative (HR+/HER2-) breast cancer. However, recommendations for endocrine therapy (ET) remained unchanged. This study examines ET trends among HR+/HER2- patients in the Netherlands and factors influencing these trends.

**Methods:**

All HR+/HER2- patients diagnosed between 2012 and 2022 who underwent surgery were selected from the Netherlands Cancer Registry. Patients were categorized by ET guideline indications: no indication; indication based on pathological, postoperative TNM-stage/grade; or indication based on clinical TNM-stage/grading on biopsy warranting neoadjuvant chemotherapy (NAC). The ET benefit was estimated for all patients using the PREDICT 2.0 tool. Logistic regression was used to identify factors associated with ET initiation.

**Results:**

The study included 127,610 HR+/HER2- patients. The proportion starting ET according to guideline recommendations decreased from 91.2 % (2012) to 79.3 % (2022), with those who did not initiate ET having a lower PREDICT-estimated benefit (1.0 %) compared to those who did (1.5 %). Reduced ET initiation was associated with age 30–39, age >80, and treatment in academic hospitals. In 2022, ET initiation varied by up to 19 percentage points across regions, while the PREDICT-estimated benefit showed no clinically relevant difference (ranging from 1.3 % to 1.5 %).

**Conclusion:**

Despite unchanged guideline recommendations, fewer patients started ET over time. This trend, and regional variation, suggests that a more reticent approach by physicians to initiating ET for HR+/HER2- breast cancer may be contributing to it.

## Highlights

• Initiating ET in HR+/HER2- patients decreased from 91 % in 2012 to 79 % in 2022, despite unchanged guideline indications.
• Patients aged 30–39 and > 80 years, and those treated in academic hospitals were less likely to start ET.
• Regional variation in ET use increased over time, with up to a19% difference, despite similar PREDICT-estimated ET benefit.
• This variation suggests that a more cautious approach to initiating ET in HR+/HER2- patients underlies the decrease in ET use.

## 1 Introduction

The introduction of adjuvant systemic therapy drastically changed breast cancer treatment [[1], [2], [3]] and contributed to improved overall survival and local control [4]. Until around 2010, the range of patients considered eligible for such adjuvant systemic therapy steadily expanded, based on consecutive international and national treatment guidelines that expanded the list of amenable conditions with lower risk breast cancers [[5], [6], [7], [8]]. Among patients with hormone receptor-positive, HER2-negative (HR+/HER2-) breast cancer, the increase in proportion of patients receiving adjuvant chemotherapy and endocrine therapy (ET) increased simultaneously [[8], [9], [10]].

After 2010, however, a countertrend emerged among patients with HR+/HER2- breast cancer. Molecular subtyping and the incorporation of gene expression profiling into clinical practice improved prognostication in luminal-type breast cancer, leading to a documented, guideline-supported decrease in chemotherapy administration. Concordantly, there was a growing apprehension that previous broadening of adjuvant systemic therapy indications had led to overtreatment [2,9,11,12]. In the meantime, clinicians have increasingly used online prognostic tools to calculate the isolated benefit of chemotherapy on overall survival. These tools have supported clinical decision making, particularly in HR+/HER2- breast cancer, and have been optimized over time. While Adjuvant! Online, which included ER status and grade as primary tumour characteristics, was routinely used at the start of the century, newer tools like PREDICT Breast 2.0 incorporate and weigh HER2 status and Ki67 expression, yielding a more refined prognostication in breast cancer patients [13,14].

In contrast to chemotherapy, the decision to initiate ET in HR+ breast cancer typically involves considering its daily treatment over a prolonged period of five to ten years. The decision to initiate ET also increasingly involves shared decision-making to weigh its potential benefits against its side effects [18], with the aforementioned prognostic tools potentially supporting this process. Recent studies are evaluating the possibility of shorter ET or even omitting ET for certain patient groups identified through gene expression profiles [19,20].

While the use of adjuvant chemotherapy has decreased, trends in ET use remain largely elusive so far [[15], [16], [17]]. This study aims to evaluate recent trends and variations in the use of adjuvant ET among Dutch HR+/HER2- breast cancer patients with an ET indication according to the national treatment guideline.

## 2 Methods

Data were obtained from the Netherlands Cancer Registry (NCR), which is a nationwide, population-based registry that includes records of all newly diagnosed malignancies, as reported by the Dutch Nationwide Pathology Database (PALGA) [21]. For this study, information on all HR+/HER2- cT1-3N0-3M0 invasive breast cancer patients diagnosed in the Netherlands between January 2012 and December 2022 was selected. Patients who did not receive upfront surgical treatment or surgery following neoadjuvant chemotherapy (NAC), were excluded. Patient, tumour, treatment, and hospital characteristics were derived from the NCR. Patients were excluded if the absence of information about tumour grade, tumour size, or lymph node status precluded the identification of an indication for ET prescription. In the present study, women under the age of 50 were classified as premenopausal, while those over 50 were classified as postmenopausal. This approach is commonly used in large breast cancer trials, such as RxPONDER and TAILORx, when menopausal status is not directly reported [22,23]. Tumour size was categorized using the cutoff values used in the treatment guidelines: <10 millimetres (mm), 10–20 mm, and >20 mm [22]. Metastatic lymph node involvement was categorized into isolated tumour cells, micrometastasis, or macrometastasis. Hospitals were categorized as academic or non-academic, based on where the surgical treatment was performed. The seven regions were defined by the NCR based on regional collaborations and comprehensive cancer organizations, with one region containing two academic centres, while all other regions contain one.

Patients were categorized as either candidates for ET or not based on the national guidelines’ indication for ET that was valid throughout the study period, which defined eligibility based on grade, tumour size, and lymph node status. For patients who underwent upfront surgery, the ET indication was based on the postoperative pathological TNM (pTNM) stage. For patients treated with NAC, the clinical assessment of tumour size and lymph node involvement (cTNM stage) formed the indication for NAC, thereby implying eligibility for adjuvant ET [24, suppl. 1].

To estimate the expected additional benefit of ET for each patient, the script of the PREDICT Breast 2.0 online tool was deployed to calculate the five-year overall survival rate with and without adjuvant ET. Then, we calculated the difference. PREDICT Breast is an online prognostic tool validated in 2011 [25,26], that supports physicians in making decisions about adjuvant therapy. Over the years the tool has been updated to include HER2 status and Ki67 expression [27,28]. To use PREDICT, we assumed that tumours in all patients aged 50–75 years were identified through the national screening program and that tumours in patients outside this age group were identified based on clinical symptoms. For this study, we estimated the standalone benefit of ET without correcting for the mitigating effect of chemotherapy use.

### 2.1 Outcomes and construction of variables

The primary outcome of our study was the proportion of patients who received ET over time, in accordance with the current guideline-based directive to initiate adjuvant ET. Among patients with a guideline-based indication for ET, the proportion who initiated ET was analysed, and in an explanatory model, we evaluated this proportion in relation to various patient, tumour, treatment, and hospital characteristics.

### 2.2 Statistical analysis

Frequencies of patient, tumour, treatment, and hospital characteristics are presented in a baseline table. The proportions of patients receiving adjuvant ET over time are reported through descriptive analyses and presented in graphs and bar charts. The median estimated expected benefit of ET is reported for patient groups categorized according to the indications for receiving ET outlined in the guidelines. A multivariable logistic regression analysis was performed to evaluate the relationship between the proportion of patients who initiated ET in accordance with the guideline recommendation and various clinicopathological, treatment, and hospital factors. Patients with unknown values for tumour size, tumour grade, lymph node status, or region, as well as those categorized as ‘other’ in type of surgery, were excluded from the multivariable analysis. Additional analyses (chi-squared test and Mann-Whitney U test) were conducted to assess whether there were substantial differences between the excluded and included patient groups. Among patients with a guideline recommendation to initiate ET, the expected benefit of ET was compared between those who did and did not initiate ET. P-values were calculated using the chi-squared test and the Kruskal-Wallis test. A p-value of less than 0.05 was regarded as statistically significant. The analyses were performed using Stata version 17.0 (StataCorp, College Station, TX) and R (version 4.4.3).

## 3 Results

### 3.1 Patients

**Fig. 1:**
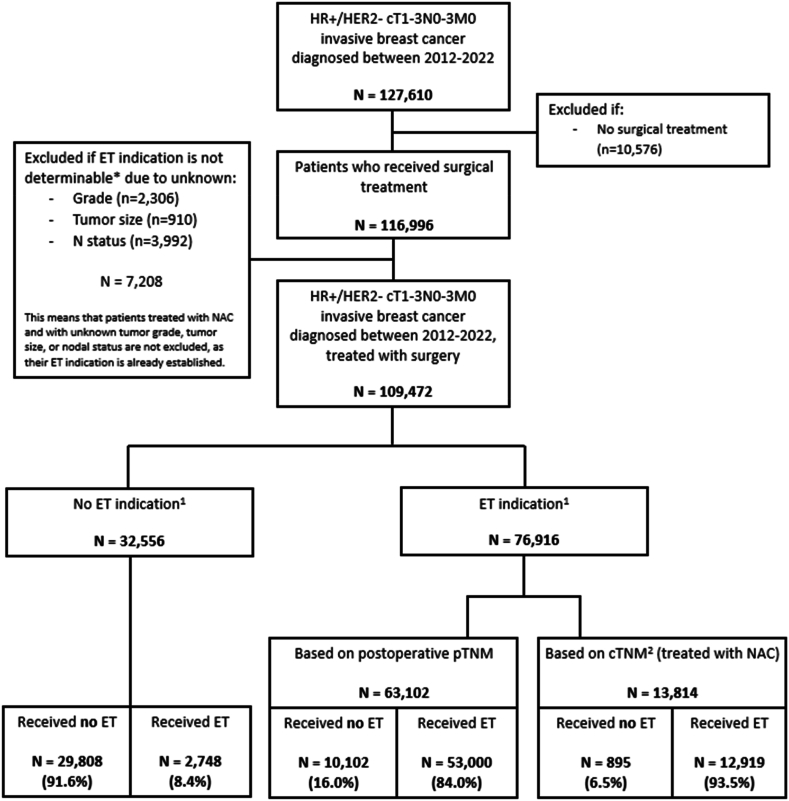
Flowchart of the study population. *HR* *+* hormone receptor-positive; *HER2-* human epidermal growth factor receptor 2-negative; *ET* endocrine therapy; *NAC* neoadjuvant chemotherapy; *TNM* tumour, nodes, metastasis; *pTNM* pathological TNM; *cTNM* clinical TNM. 1.Tumours with an indication for adjuvant endocrine therapy according to the Dutch treatment guidelines. - Grade 1, >2 cm and N0 (node-negative or isolated tumour cells). - Grade 1, all sizes and N+ (node-positive, i.e., micrometastasis or macrometastasis). - Grade 2 or grade 3, >1 cm and N0 (node-negative or isolated tumour cells). - Grade 2 or grade 3, all sizes and N+ (node-positive, i.e., micrometastasis or macrometastasis). 2. Based on biopsy.

A total of 127,610 patients with newly diagnosed cT1-3N0-3M0 HR+/HER2- breast cancer who were treated between 2012 and 2022 were identified in the NCR. Of these patients, 116,996 underwent surgical treatment. Due to missing data on tumour grade, tumour size, or lymph node status, precluding the identification of an indication for prescribing ET, 7208 patients were excluded (Fig. 1). Of the remaining 109,472 patients, 32,556 had no indication for ET and 63,102 did have an ET indication after upfront surgical treatment, while 13,814 were initially treated with NAC and had an inherent postoperative ET indication.

Over the years, the proportion of patients eligible for adjuvant ET according to Dutch treatment guidelines remained stable (approximately 70 %). In this group with an ET indication, the proportion of patients treated with NAC increased from 10.9 % in 2012 to 21.4 % in 2022. Over time, the average age of our study population increased by approximately 1 year. The median expected benefit of ET as calculated by PREDICT was a 0.4 % percent lower risk of death after five years in the group of patients without an ET indication, a 1.3 % lower risk in patients with an ET indication based on pTNM (after upfront surgery), and a 1.9 % lower risk in patients who were treated with NAC (Table 1).

**Table 1:** Baseline characteristics of all HR+/HER2- breast cancer patients treated with surgery.

	N overall (N = 109,472)	No ET indication (N = 32,556)		ET indication based on pTNM (N = 63,102)		ET indication based on cTNM and treated with NAC (N = 13,814)	
	*N*	*N*	*%*	*N*	*%*	*N*	*%*
**Year of incidence**							
2012	9519	2885	*30*	5911	*62*	723	*8*
2013	10,020	3017	*30*	6032	*60*	971	*10*
2014	9806	2903	*30*	5829	*59*	1074	*11*
2015	9972	2960	*30*	5574	*56*	1438	*14*
2016	10,192	3032	*30*	5945	*58*	1215	*12*
2017	10,345	3186	*31*	5940	*57*	1215	*12*
2018	9930	3008	*30*	5610	*57*	1312	*13*
2019	9994	2957	*30*	5610	*56*	1427	*14*
2020	8480	2208	*26*	4983	*59*	1289	*15*
2021	10,582	3144	*30*	5869	*55*	1569	*15*
2022	10,632	3256	*31*	5799	*54*	1577	*15*
**Age category (year)**							
<30	319	40	*13*	109	*34*	170	*53*
30–39	3314	487	*15*	1336	*40*	1491	*45*
40–49	15,580	2898	*19*	8118	*52*	4564	*29*
50–59	27,093	8383	*31*	14,575	*54*	4135	*15*
60–69	32,241	11,571	*36*	17,801	*55*	2869	*9*
70–79	22,494	7837	*35*	14,084	*63*	572	*2*
>80	8431	1340	*16*	7079	*84*	12	*0*
**Menopausal status**							
Premenopausal	20,514	4010	*19*	10,180	*50*	6324	*31*
Perimenopausal	3972	1101	*28*	2156	*54*	715	*18*
Postmenopausal	84,986	27,445	*32*	50,766	*60*	6775	*8*
**Tumour size (mm)**							
<10	24,878	19,427	*78*	2034	*8*	3417	*14*
10–20	50,784	13,129	*26*	33,696	*66*	3959	*8*
>20	33,387	0	*0*	27,372	*82*	6015	*18*
Unknown	423	–	*-*	–	*-*	423	*100*
**Tumour grade**							
Grade 1	31,828	21,771	*69*	8711	*27*	1346	*4*
Grade 2	58,911	9590	*16*	42,356	*72*	6965	*12*
Grade 3	16,231	1195	*7*	12,035	*74*	3001	*19*
Unknown	2502	–	*-*	–	*-*	2502	*100*
**Pathological N stage**							
Negative	69,896	31,289	*45*	33,699	*48*	4908	*7*
Isolated tumour cells	5564	1267	*23*	3654	*66*	643	*12*
Micrometastasis	8840	0	*0*	7599	*86*	1214	*14*
Macrometastasis	24,788	0	*0*	18,150	*73*	6638	*27*
Unknown	384	–	*-*	–	*-*	384	*100*
**Estimated ET benefit using PREDICT∗** (median (IQR))	1.0 (0.5–1.9)	0.4 (0.3–0.5)		1.3 (0.9–2.3)		1.9 (1.1–3.4)	
**Type of surgery**							
Lumpectomy	73,696	26,912	*36*	39,623	*54*	7161	*10*
Ablatio	35,744	5638	*16*	23,464	*66*	6642	*18*
Other	32	6	*19*	15	*47*	11	*34*
**Chemotherapy**							
No	78,246	32,234	*41*	46,012	*59*	–	*-*
Neoadjuvant	13,814	–	*-*	–	*-*	13,814	*100*
Adjuvant	17,412	322	*2*	17,090	*98*	–	*-*
**Type of hospital (surgery)**							
Non-academic	101,147	30,328	*30*	58,817	*58*	12,002	*12*
Academic	8325	2228	*27*	4285	*51*	1812	*22*
**Region (surgery)**							
1	21,625	6129	*28*	11,825	*55*	3671	*17*
2	15,140	4148	*27*	9390	*62*	1602	*11*
3	10,364	3131	*30*	5980	*58*	1253	*12*
4	9515	2875	*30*	5380	*57*	1260	*13*
5	18,169	5470	*30*	10,456	*58*	2243	*12*
6	22,359	6776	*30*	13,373	*60*	2210	*10*
7	12,216	4010	*33*	6650	*54*	1556	*13*
Unknown	84	17	*20*	48	*57*	19	*23*

*HR +* hormone receptor-positive; *HER2-* human epidermal growth factor receptor 2-negativee; *ET* endocrine therapy; *TNM* tumour, nodes, metastasis; *pTNM* pathological TNM; *cTNM* clinical TNM; *NAC* neoadjuvant chemotherapy; *mm* millimetre; *IQR* interquartile range.

∗ The reported benefit pertains to the 5-year overall survival. Due to missing data precluding estimation of the additional ET benefit, 5960 patients were excluded from this analysis.

Of all patients eligible for ET according to the national guidelines 85.7 % received ET, of whom 93.8 % received it in the adjuvant setting and 6.0 % received it in the neoadjuvant as well as in the adjuvant setting. The remaining 0.2 % of patients received neoadjuvant ET but did not proceed with adjuvant ET, as originally intended. Among patients receiving ET, the proportion treated in the neoadjuvant setting increased from 1.5 % to 13.4 %. While 84.0 % of the patients with an indication after upfront surgery and 93.5 % of the patients treated with NAC received ET, eight percent of patients without a guideline indication received ET (Fig. 1).

### 3.2 Adjuvant ET over time and regional variation

**Fig. 2:**
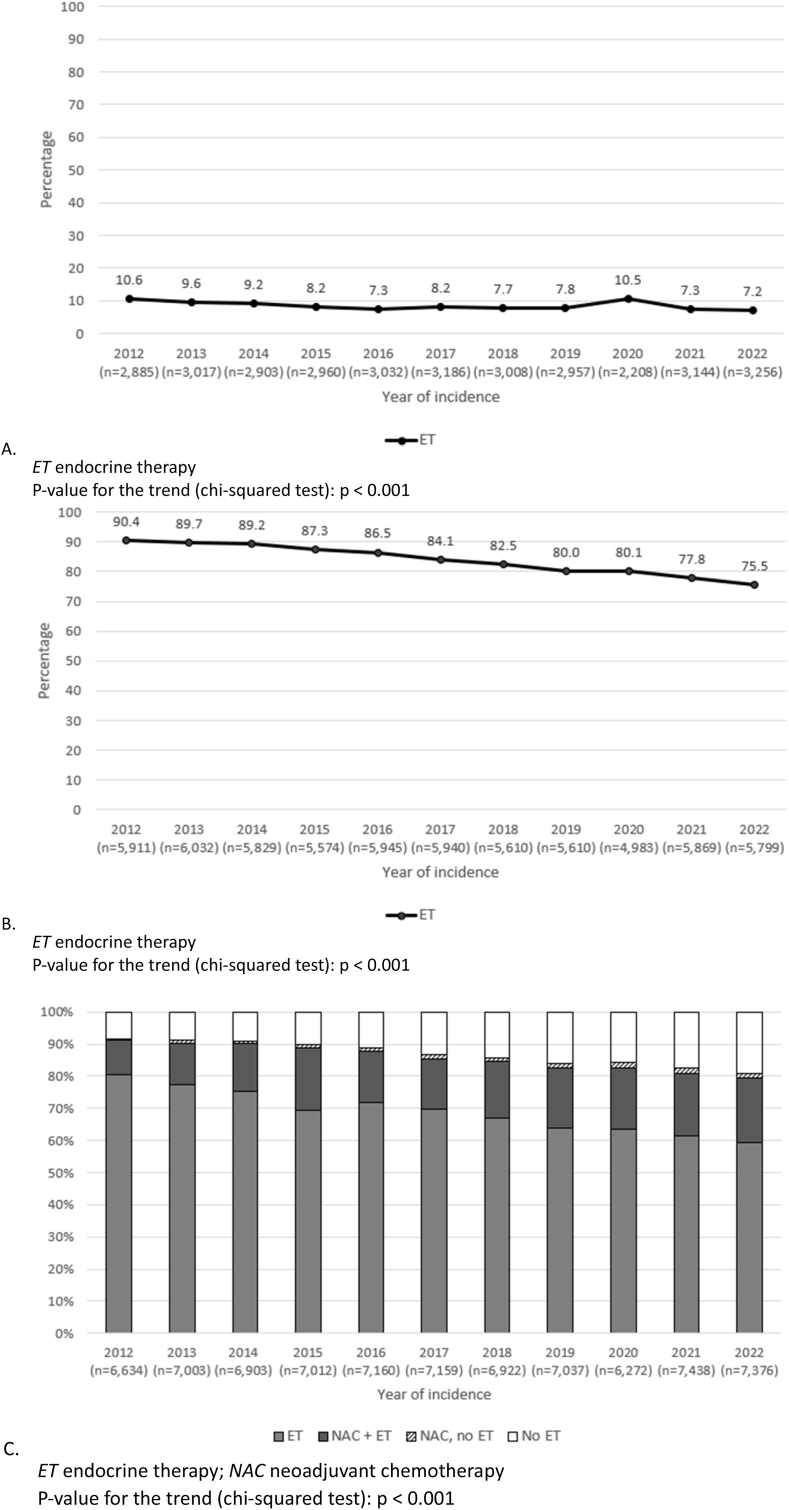
The proportion of patients receiving adjuvant ET from 2012 to 2022, in (A) patients without an ET indication, (B) patients with an ET indication based on postoperative pTNM and (C) patients with an ET indication based on postoperative pTNM and cTNM (those who were treated with NAC).

**Fig. 3:**
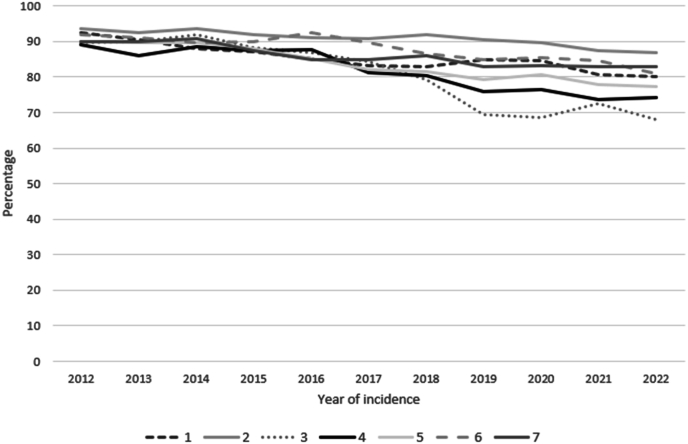
The proportion of patients with an ET indication (including those treated with NAC) receiving adjuvant ET by region∗ from 2012 to 2022 (N = 76,849) ∗ Region ‘unknown’ is excluded from the figure (n = 73). *ET* endocrine therapy; *NAC* neoadjuvant chemotherapy. P-value for the trend per region (chi-squared test): 1: ref; 2: p = 0.338; 3: p < 0.001; 4: p = 0.016; 5: p = 0.069; 6: p = 0.619; 7: p = 0.164.

Overall, the proportion of patients receiving adjuvant ET decreased from 66.8 % in 2012 to 57.2 % in 2022. In patients with no ET indication, the proportion who received adjuvant ET decreased from 10.6 % to 7.2 % (Fig. 2a). In patients with an ET indication after primary surgery a decrease from 90.4 % to 75.5 % was observed (Fig. 2b), and in patients treated with NAC a decrease from 97.8 % to 93.3 % was observed. In all patients with an ET indication according to the national guideline (n = 76,916), the proportion of patients receiving adjuvant ET decreased from 91.2 % to 79.3 % (Fig. 2c). In 2012, the proportion of patients receiving ET ranged from 89.0 % to 93.5 % across the seven NCR-defined regions. In 2022 the variation ranged from 67.9 % to 86.8 %, representing a nearly 20 percentage point gap (Fig. 3). These differences were also reflected in the variation in odds ratio between the regions in the multivariable logistic regression analysis (Table 2). Across the NCR regions, the mean expected benefit of ET as calculated by PREDICT on 5-year overall survival ranged from 1.3 % to 1.5 % (Kruskal-Wallis test: p < 0.001). Younger patients were more often treated at an academic centre. Given the equal distribution of one academic centre per region (except for one region, which contains two), no clinically relevant age differences between regions was observed.

**Table 2:** Multivariable logistic regression analysis to evaluate association of patient, tumour, treatment, and hospital characteristics with initiating ET in patients with an ET indication according to the treatment guidelines.

	N total = 73,765^a^		Multivariable analysis	
	N	% ET	Odds ratio	95 % CI
**Age categories**				
<30	249	90.8	0.86	0.55–1.34
30–39	2484	88.8	**0.70**	**0.60–0.81**
40–49	11,541	91.0	Ref	
50–59	17,760	88.5	1.09	0.96–1.24
60–69	20,086	86.6	1.10	0.95–1.26
70–79	14,555	81.9	0.87	0.75–1.00
>80	7090	70.4	**0.39**	**0.34–0.45**
**Menopausal status**				
Premenopausal	14,991	90.5	Ref	
Perimenopausal	2678	90.7	1.04	0.88–1.22
Postmenopausal	56,096	83.7	**0.82**	**0.73–0.94**
**Tumour size (mm)**				
<10	4770	84.4	Ref	
10–20	37,056	83.2	**1.69**	**1.53–1.86**
>20	31,939	87.9	**2.38**	**2.15–2.63**
**Tumour grade**				
Grade 1	9990	81.0	Ref	
Grade 2	48,886	85.2	**1.74**	**1.63–1.85**
Grade 3	14,889	88.8	**1.97**	**1.82–2.13**
**Pathological N stage**				
Negative	37,606	80.4	Ref	
Isolated tumour cells	4172	86.5	**1.44**	**1.31–1.59**
Micrometastasis	8603	84.6	**1.51**	**1.41–1.62**
Macrometastasis	23,384	93.4	**3.11**	**2.93–3.13**
**Type of surgery**				
Lumpectomy	45,156	84.3	Ref	
Ablatio	28,609	87.0	**1.06**	**1.01–1.11**
**Chemotherapy**				
None	45,988	80.0	Ref	
Neo-adjuvant	10,714	93.3	**2.65**	**2.41–2.92**
Adjuvant	17,063	94.7	**2.49**	**2.30–2.70**
**Type of hospital (surgery)**				
Non-academic	68,118	85.6	Ref	
Academic	5647	82.8	**0.70**	**0.65–0.76**
**Region (surgery)**				
1	14,534	84.7	Ref	
2	10,699	90.8	**1.69**	**1.55–1.83**
3	6892	80.3	**0.72**	**0.66–0.77**
4	6331	81.0	**0.69**	**0.64–0.75**
5	12,167	83.1	**0.85**	**0.79–0.91**
6	15,161	87.8	**1.26**	**1.18–1.36**
7	7981	85.7	**1.14**	**1.05–1.24**

*ET* endocrine therapy; *CI* confidence interval; *mm* millimetre.

a Patients with unknown values for tumour size (n = 423), tumour grade (n = 2401), lymph node status (n = 268), or region (n = 40) and patients in the ‘other’ category from type of surgery (n = 19) were excluded from the multivariable logistic regression analysis.

### 3.3 The relation with patient, tumour, treatment, and hospital characteristics

A total of 3151 patients were excluded from the multivariable test due to unknown values for tumour size, tumour grade, lymph node status, or region, as well as those categorized as ‘other’ in type of surgery. There was no significant difference in baseline characteristics between the included and excluded patients. Apart from the trend over time and the regional differences, factors associated with less ET use were age 30–39 years and age >80 years, postmenopausal status, and treatment in an academic hospital (see Table 2). Factors associated with a higher chance of receiving adjuvant ET included larger tumour size, higher tumour grade, and increasing lymph node involvement. Patients treated with chemotherapy (neoadjuvant as well as adjuvant) also had an increased chance of receiving adjuvant ET compared to patients not treated with chemotherapy. Among patients with an ET indication according to the national guideline (including those treated with NAC), the median expected benefit of ET as calculated by PREDICT was a 1.5 % improved OS in patients who started ET, compared to 1.0 % in those who did not (Kruskal-Wallis test: p < 0.001).

## 4 Discussion

In this population-based study, a decreasing trend in ET use was observed among HR+/HER2- breast cancer patients for whom treatment with ET was recommended according to national guidelines. The proportion of patients initiating ET decreased significantly over the ten-year study period. Various patient, tumour, and treatment factors were associated with ET use, and the trend varied significantly across regions, regardless of the estimated benefit of ET use according to PREDICT.

Overall, 86 % of patients who were recommended to initiate ET based on guideline recommendations did so during the study period. This proportion aligns with the findings of a European systematic review, which reported an overall guideline adherence rate for ET of 90 % [29]. One American study published in 2013 reports that 89 % of patients used ET [30], while another American study describes that 70 %–80 % of patients with an ET indication initiated ET [31]. Moreover, the adherence rate for ET is comparable to the rates for surgical procedures, chemotherapy, and radiotherapy for breast cancer [29].

During the study period, we observed a more than 10 % decrease in the number of patients receiving adjuvant ET, despite the unchanged indications for ET in treatment guidelines. This decline is substantial and clinically relevant, with some regions showing an even larger decrease. The more cautious approach to initiating ET is supported by the median expected benefit of ET as calculated by PREDICT: a 1.5 % improved OS in patients who started ET, compared to 1.0 % in those who did not.

A U.S. study showed a different trend: an increase in women initiating ET, from 81 % in 2002 to 88 % in 2014. This increase was attributed to the introduction of aromatase inhibitors. Subsequently, there was a decrease in the proportion of patients initiating ET, reaching 83 % [32]. To our knowledge, no data are available on time trends in the initiation of ET among European patients who are eligible according to guidelines. While the group of patients eligible for adjuvant ET remained stable during the study period, treatment guidelines have narrowed the group of patients eligible for adjuvant chemotherapy over the past decade [24]. In the same cohort of HR+/HER2- patients and timeframe, adjuvant chemotherapy was administered to fewer patients, partly due to the use of gene expression profiles [[15], [16], [17]]. Furthermore, among patients eligible for ET, the proportion receiving neoadjuvant ET (NET) increased, reflecting the evolving perspective on NET over the past decade [33,34], and being influenced by the COVID-19 pandemic [35]. Over the past decade, the role of NET in HR+/HER2- breast cancer has expanded beyond its original application in frail or elderly patients deemed unfit for chemotherapy. There is increasing evidence that NET can effectively downstage tumours and axillary lymph node disease, improving the likelihood of breast-conserving surgery and allowing for less extensive local therapy. Moreover, NET has gained clinical relevance as a bridge to surgery, especially during the COVID-19 pandemic, as well as a means of assessing treatment response and investigating predictive biomarkers. These developments have shifted the perception of NET from a niche option for a select group of patients to a widely applicable, evidence-based treatment strategy.

The proportion of patients who receive ET is associated with several tumour and patient characteristics. Patients with larger tumours, higher tumour grades, or lymph node involvement were more likely to receive ET. Patients were also more likely to receive ET if they underwent a mastectomy or were treated with chemotherapy. A lower proportion of patients received ET if they were older (i.e., >80 years of age), were between 30 and 39 years old, or postmenopausal. It must be considered that for patients over 80 years of age, other factors may influence the decision to withhold ET, such as comorbidities and competing risks. Other studies have shown that age, tumour size, tumour grade, and lymph node involvement are also associated with an increased or decreased administration of chemotherapy [9,15,16]. Furthermore, extremes of age were significantly associated with persistent ET use as well [30,36,37]. Other studies have demonstrated that initiation of ET is associated with receiving other types of treatment, the presence of diabetes, race [31], worry about recurrence, and inadequate provision of information about possible side effects [30]. Our study shows that the location where a patient was treated was also associated with ET initiation. We found that patients treated at an academic centre were less likely to initiate ET. Furthermore, the decreasing proportion of patients who started ET over time varied substantially by region. By the end of the study period, the proportion of patients who started ET ranged from 68 % to 87 % across regions. While there were statistically significant differences in the expected benefit of ET between regions, these differences were clinically irrelevant. They reflect minimal variation in baseline risk between regions, while there was no linear relationship between this PREDICT-based estimation of ET benefit and the proportion of patients receiving ET in a region. The distribution of the patient and tumour characteristics remained stable over time, except for a modest increase in median age. Additionally, the proportion of all patients who were eligible for ET use remained consistent over time. Most of the patient and tumour characteristics associated with ET use are conceivable for their documented effect on recurrent disease and the inherent expected benefit of ET. The absence of such a relationship for the observed differences between the regions and the variation in ET use across regions over time suggests that the estimated benefit of ET is weighed differently in different regions.

The overall decrease in ET use is likely due to a changing perception of the risk of recurrent breast cancer and the inherent benefit of ET. A more cautious approach to administering chemotherapy to the same patient population, partly guided by gene expression profiles, has likely contributed to the decreasing trend by altering physicians' expectations regarding the benefits of ET. Shared decision-making processes have also gained more attention in the past two decades and have been enhanced by online prognostic tools such as PREDICT Breast. While patients have become more assertive in shared decision-making over the past decade [38,39], it is likely that physician attitudes and the shared decision-making process explain the overall decrease in ET use. Due to prognostic tools, physicians can now discuss the calculated survival benefits of adjuvant systemic therapy in relation to an individual patient's life expectancy and better weigh the estimated effects against the side effects of chemotherapy and ET. This study's observation that patients who did not initiate ET, despite a guideline recommendation, had a lower expected benefit of ET than those prescribed ET strongly supports this view. Furthermore, the median expected benefit of ET as calculated by PREDICT was overall low. In this respect, it is also important to note that in the Netherlands there is a more cautious approach to adjuvant ET compared to other countries, as shown by randomised trials on axillary treatment. In the BOOG2013-08 trial, a substantially lower proportion of patients received ET compared with the international SOUND and INSEMA trials [[40], [41], [42]]. We believe that further research into the motivations of both physicians and patients, as well as studies on long-term outcomes, is needed to determine whether the more cautious approach to adjuvant ET is justified.

This is a large, population-based study that uses national cancer registry data. The study period spans twelve years, beginning in the year that the last expansion of the group of patients eligible for adjuvant systemic therapy was included in the guidelines (2012). There are several limitations, partly due to the retrospective nature of the study and the registry's limited scope. In line with the research question, the data includes information on the initiation of adjuvant ET, but not on persistence with or adherence to therapy. It should be noted that up to 40 % of patients discontinue medication early, and 30 % take medication less frequently than prescribed [36,37]. Adherence is associated with adverse effects, numerous demographic and clinical factors, and patient-reported personal and social factors [36,37,43]. Additionally, this study is limited by the lack of information regarding the decision-making process. It is unclear what was recommended during the multidisciplinary team meetings, which healthcare provider (surgeon, oncologist, or specialized nurse) communicated with patients, and if PREDICT's calculated additional ET benefit and survival estimates were discussed. Furthermore, there is no information available about the motivation and reasoning of patients and physicians to start or refrain from ET. These factors should be investigated in a different type of study that includes questionnaires and interviews with physicians and patients to clarify the decision-making process. Lastly, this study slightly overestimated the additional ET benefit because the PREDICT calculations were based on the assumption that patients had not received other adjuvant therapies, which would diminish the estimated standalone effect of ET.

In conclusion, the initiation of adjuvant ET for HR+/HER2- breast cancer patients in the Netherlands significantly decreased between 2012 and 2022, despite unchanged guideline indications. Key factors associated with initiating ET were younger age, higher tumour grade, larger tumour size, lymph node involvement, and chemotherapy treatment. Geographical differences likely reflect shifts in physicians’ attitudes toward personalized treatment strategies. These findings underscore the growing influence of shared decision-making in balancing the benefits and side effects of therapy, emphasizing the need for further research into patient and physician decision-making processes, as well as long-term outcomes.

## CRediT authorship contribution statement

**Eline E.F. Verreck:** Writing – original draft, Visualization, Formal analysis, Conceptualization. **Emily L. Postma:** Writing – review & editing. **Tanja Oostergo:** Writing – review & editing. **Joyce Meijer:** Writing – review & editing. **Anouk Eijkelboom:** Writing – review & editing. **Sabine Siesling:** Writing – review & editing. **Dimitris Rizopoulos:** Writing – review & editing, Formal analysis. **Thijs van Dalen:** Writing – original draft, Supervision, Methodology, Conceptualization. **José H. Volders:** Writing – original draft, Supervision, Methodology, Conceptualization.

## Funding

The research is partly funded by Diakonessenhuis research grant 2024.

## Declaration of competing interest

The authors declare that they have no known competing financial interests or personal relationships that could have appeared to influence the work reported in this paper.

## Data Availability

Datasets described and analysed in this manuscript are available from the corresponding author on reasonable request.
