# Supplementary material for: Less endocrine therapy in HR+/HER2- breast cancer: a nationwide trend despite unchanged guidelines
Source: Breast. 2025 Nov 26;85:104664. doi: 10.1016/j.breast.2025.104664 (PMC12719210; doi:10.1016/j.breast.2025.104664)
Supplement: Multimedia component 1 [file mmc1.docx]

**Appendix**

**Supplementary figure 1:** Endocrine therapy according to the Dutch treatment guidelines [1]

1. Federation of Medical Specialists. Treatment guideline breast cancer ‘endocrine therapy’, PDF from 12-03-2021, last authorized 07-02-2020 [Internet]. Available from: [Borstkanker - Endocriene therapie - Richtlijn - Richtlijnendatabase](https://richtlijnendatabase.nl/richtlijn/borstkanker/behandeling_invasief_carcinoom/adjuvante_systemische_behandeling/endocriene_therapie.html). [Accessed 21^st^ July 2025].

**Indications for (neo)adjuvant endocrine therapy**

If ER > 10% and/or PR > 10%, regardless of age, HER2 status, and orange text

| **Differentiation grade** | **Tumour diameter** | N0/N0(i+) | N1mi/N1-3 |
| --- | --- | --- | --- |
| Grade 1 | ≤ 1 cm | Green | Orange |
|  | 1.1-2 cm | Green | Orange |
|  | 2.1-5 cm | Orange | Orange |
|  | > 5 cm | Orange | Orange |
|  |  |  |  |
| Grade 2 | ≤ 1 cm | Green | Orange |
|  | 1.1-2 cm | Orange | Orange |
|  | 2.1-5 cm | Orange | Orange |
|  | > 5 cm | Orange | Orange |
|  |  |  |  |
| Grade 3 | ≤ 1 cm | Green | Orange |
|  | 1.1-2 cm | Orange | Orange |
|  | 2.1-5 cm | Orange | Orange |
|  | > 5 cm | Orange | Orange |

*N0(i+)* isolated tumour cells; *N1mi* micrometastasis; *cm* centimetre

Orange: Endocrine therapy indicated

Green: Endocrine therapy not indicated

**Indications for (neo)adjuvant endocrine therapy based on tumour type, tumour size, and lymph node status.**

| **Tumour type**  **Tumour grading** | **Tumour size** | **Lymph node status** | **Adjuvant regimen** |
| --- | --- | --- | --- |
| ER+ HER2+/- |  |  |  |
| Grade 1 | > 2 cm | N0 | * |
|  | any | N+ | > 5 years |
| Grade 2 | > 1 cm | N0 | * |
|  | any | N+ | > 5 years |
| Grade 1 | > 1 cm | N0 | * |
|  | any | N+ | > 5 years |

*ER+ o*estrogen receptor-positive; *HER2+/-* human epidermal growth factor receptor 2-positive/negative; *cm* centimetre; *N+* node-positive (micrometastasis/macrometastasis)

See also module Adjuvant endocrine therapy

*) Consider > 5 years in high-risk breast cancer
